# Investigating the Relationship between White Matter Connectivity and Motivational Circuits in Subjects with Deficit Schizophrenia: A Diffusion Tensor Imaging (DTI) Study

**DOI:** 10.3390/jcm11010061

**Published:** 2021-12-23

**Authors:** Giulia M. Giordano, Pasquale Pezzella, Mario Quarantelli, Paola Bucci, Anna Prinster, Andrea Soricelli, Andrea Perrottelli, Luigi Giuliani, Michele Fabrazzo, Silvana Galderisi

**Affiliations:** 1 Department of Psychiatry, University of Campania “Luigi Vanvitelli”, 80138 Naples, Italy; pezzella.pasquale3@gmail.com (P.P.); paolabucci456@gmail.com (P.B.); andreaperrottelli@gmail.com (A.P.); luigi.giuliani.91@gmail.com (L.G.); michele.fabrazzo@unicampania.it (M.F.); silvana.galderisi@gmail.com (S.G.); 2 Biostructure and Bioimaging Institute, National Research Council, 80134 Naples, Italy; quarante@unina.it (M.Q.); anna.prinster@ibb.cnr.it (A.P.); 3 Department of Integrated Imaging, IRCCS SDN, 80143 Naples, Italy; andrea.soricelli@uniparthenope.it; 4 Department of Motor Sciences and Healthiness, University of Naples Parthenope, 80133 Naples, Italy

**Keywords:** motivation circuits, negative symptoms, RDoC, positive valence system, salience system, schizophrenia, deficit syndrome

## Abstract

Deficit schizophrenia is a subtype of schizophrenia presenting primary and enduring negative symptoms (NS). Although one of the most updated hypotheses indicates a relationship between NS and impaired motivation, only a few studies have investigated abnormalities of motivational circuits in subjects with deficit schizophrenia (DS). Our aim was to investigate structural connectivity within motivational circuits in DS. We analyzed diffusion tensor imaging (DTI) data from 46 subjects with schizophrenia (SCZ) and 35 healthy controls (HCs). SCZ were classified as DS (*n* = 9) and non-deficit (NDS) (*n* = 37) using the Schedule for Deficit Syndrome. The connectivity index (CI) and the Fractional Anisotropy (FA) of the connections between selected brain areas involved in motivational circuits were examined. DS, as compared with NDS and HCs, showed increased CI between the right amygdala and dorsal anterior insular cortex and increased FA of the pathway connecting the left nucleus accumbens with the posterior insular cortex. Our results support previous evidence of distinct neurobiological alterations underlying different clinical subtypes of schizophrenia. DS, as compared with NDS and HCs, may present an altered pruning process (consistent with the hyperconnectivity) in cerebral regions involved in updating the stimulus value to guide goal-directed behavior.

## 1. Introduction

Negative symptoms represent a core aspect of schizophrenia, with a negative impact on the functioning of people suffering from this disorder. To date, they remain an unmet therapeutic need, since no effective treatment is available for these symptoms, particularly when they are primary to the disorder [1,2,3,4,5,6,7,8,9,10,11,12,13,14]. 

According to the current conceptualization provided by the Consensus Conference of the National Institute of Mental Health—Measurement and Treatment Research to Improve Cognition in Schizophrenia (NIMH-MATRICS), the negative symptom construct includes five individual symptoms, namely avolition, anhedonia, asociality, blunted affect and alogia [15]. These symptoms cluster into two domains, the Experiential domain (which includes avolition, anhedonia and asociality) and the Expressive Deficit domain (which includes blunted affect and alogia) [4,13,14,15,16,17,18,19,20]. 

Negative symptoms might be the primary manifestation of schizophrenia (primary negative symptoms) or the consequence of different factors (secondary negative symptoms), i.e., psychopathological factors (moderate positive symptoms, clinically significant depression), syndrome-unrelated factors (social isolation, environmental hypostimulation) or medication side effects (extrapyramidal symptoms and sedation), and might be transient or persistent over time. Primary and persistent negative symptoms characterize a subtype of schizophrenia, named deficit schizophrenia, which is associated with a greater impairment of general cognitive functions and poorer treatment response and outcome, in comparison with non-deficit schizophrenia [21,22,23,24,25,26,27,28,29,30].

One of the most updated neurobiological hypotheses underlying negative symptoms indicates a relationship between the Experiential domain and an impairment in different aspects of motivation [4,27,31,32,33,34,35,36,37,38,39,40,41,42]. Indeed, subjects with schizophrenia show impairments in several aspects of motivation, except for the pleasure experience [31,32,34,35,36]. Notably, patients show greater difficulty in reward-related learning and adaptive integration of value information with action selection [43,44], which could be linked to an alteration of the connectivity between brain areas involved in the dopaminergic circuits. On the other hand, the Expressive Deficit domain is less understood and probably is related to deficits in neurocognitive and social cognition abilities—often observed in subjects with schizophrenia, particularly in subjects with a high genetic risk for schizophrenia [5,7,45,46,47,48,49,50,51]—and to neurological soft signs, suggesting that Expressive Deficit symptoms, akin to cognitive deficits, are probably driven by a diffuse neurodevelopmental disconnectivity [4,52,53]. 

Two possible mechanisms and circuits might be implicated in the pathophysiology of motivational deficits in subjects with schizophrenia: an impairment in the “motivational value system or reward circuit” (NIMH Research Domain Criteria “positive valence system”) and/or an impairment in the “motivational salience circuit”. The brain areas belonging to the motivational value system are the ventral tegmental area (VTA) and the ventro-medial substantia nigra pars compacta (VMSNpc), which project to the nucleus accumbens shell (sNAcc), the dorsal striatum (DStr), the medial orbito-frontal cortex (mOFC) and the ventro-medial prefrontal cortex (VMPFC) [4,33,39]. Abnormalities in these areas and/or in their connections may result in an impairment in anticipatory pleasure, action evaluation and encoding of the value of stimuli, action outcome contingency learning (the ability to know the causal consequences of an action) and instrumental learning (the integration of value with action selection) [4].

The motivational salience system includes the VTA and the dorso-lateral substantia nigra pars compacta (DLSNpc) with projections to the accumbens core (cNAcc), which, in turn, projects to the DSr, the dorso-lateral prefrontal cortex (DLPFC), the ventro-lateral prefrontal cortex (VLPFC) and the anterior cingulate cortex (ACC) [33]. Abnormalities in these areas and/or in their connections might lead to an impairment in general and energetic aspects of motivation, vigor in motivated behavior, cognitive activation and the ability to orient oneself towards salient stimuli [4,33,54,55,56,57]. The identification of biobehavioral data associated with specific psychopathological features might refine hypotheses on negative symptoms [58], clarify the relationships with cognitive impairment and pave the way towards innovative treatment options for some of these symptoms [59].

Although several brain regions are part of these two interconnected circuits (motivational value and salience systems), the nucleus accumbens (NAcc) and the VTA represent key central regions within these circuits [60,61]. Other brain structures interconnected with these circuits are the amygdala (Amy) and hippocampus [62,63]. 

As far as we know, only rarely have these pathophysiological models of negative symptoms been applied to the deficit schizophrenia construct [4,37,64]. In particular, one study [64] reported the presence in subjects with deficit schizophrenia of structural brain abnormalities in several brain areas, such as the insula, anterior cingulate cortex, medial prefrontal cortex and putamen, which are involved in motivation and goal-directed behavior. In a functional magnetic resonance imaging study during a reward anticipation task, Mucci and colleagues [37] reported that subjects with deficit schizophrenia showed a significant reduction in dorsal caudate activity, compared with both healthy controls and subjects with non-deficit schizophrenia. 

Diffusion tensor imaging (DTI) studies have highlighted the presence of “disconnectivity” within and between cortical and subcortical areas in subjects with schizophrenia and in those with psychotic disorders [37,39,40,65,66,67,68,69,70,71,72,73]. This disconnectivity might lead to abnormalities in those pathways that underlie cognitive abilities and motivated behavior [65,74]. 

In subjects with deficit schizophrenia, white matter (WM) abnormalities in the superior longitudinal fasciculus [75], left uncinate fasciculus [76,77], right inferior longitudinal fasciculus, right arcuate fasciculus [77], postcentral area, left forceps minor [78], right posterior thalamic radiation [79] and posterior corpus callosum [80] have been reported. 

However, these studies did not investigate abnormalities of motivational circuits in subjects with deficit schizophrenia, since this was not the primary objective of these studies. Furthermore, some of the above-mentioned studies [77,79,80] did not use the Schedule for the Deficit Syndrome (SDS), which represents the gold standard to assess deficit schizophrenia, but they instead used a proxy from the Positive and Negative Syndrome Scale (PANSS) [81]. However, it has been demonstrated that the proxy for categorizing patients in subjects with deficit and non-deficit schizophrenia has some problems in terms of face validity and temporal stability [14]. In addition, the PANSS includes some aspects that are not conceptualized as negative symptoms and evaluates symptoms belonging to the Experiential domain only at a behavioral level. 

Therefore, in light of the above observations, our study aimed to fill the gap in the previous literature, investigating, in subjects with deficit schizophrenia (assessed with a state-of-the-art instrument), the presence of abnormalities within motivational circuits. To this aim, using a bilateral probabilistic approach on DTI data, the present study examined differences between subjects with deficit schizophrenia, subjects with non-deficit schizophrenia and healthy controls in WM connections between major brain regions involved in motivational pathways. We hypothesized that subjects with deficit schizophrenia would show abnormalities in WM connections between brain areas involved in motivational circuits, compared to subjects with non-deficit schizophrenia and healthy controls.

## 2. Materials and Methods

### 2.1. Subjects

Fifty-two subjects with schizophrenia (SCZ) were enrolled at the Department of Psychiatry of the University of Campania “Luigi Vanvitelli”, in the period between September 2010 and July 2012. All subjects were right-handed. 

The inclusion criteria were as follows:

- diagnosis of schizophrenia based on the criteria of the DSM-IV, confirmed by the Mini International Neuropsychiatric Interview Plus (MINI-Plus);
- age between 18 and 65 years;
- negative history of intellectual disability, head trauma with unconsciousness, alcohol or substance abuse within the previous six months (except for cigarette smoking);
- no treatment modifications and/or hospitalization due to symptom exacerbation in the last three months;
- treatment with second-generation antipsychotics [82].

Thirty-five right-handed healthy controls (HCs) were included. The subjects were enrolled from the community through the distribution of informative leaflets. Exclusion criteria for HCs were:

- presence of current or lifetime Axis I or II psychiatric diagnosis; history of psychiatric hospitalization;
- history of head trauma with unconsciousness;
- history of substance abuse or dependence (except for cigarette smoking) and use of drugs that affect the central nervous system.

The study was approved by the University Ethics Committee. All participants signed a written informed consent form after a detailed description of the study procedures and goals.

The study was performed in accordance with the ethical principles of the Declaration of Helsinki. 

A subsample of thirty-five SCZ and seventeen HCs was included in a previous publication [40].

### 2.2. Assessment Instruments

Socio-demographic variables such as age, paternal and maternal education and gender were evaluated for all subjects. A semi-structured interview, the Schedule for the Deficit Syndrome [83], was used to categorize patients as subjects with deficit schizophrenia (DS) and subjects with non-deficit schizophrenia (NDS). In particular, deficit schizophrenia was diagnosed when subjects had at least two out of six primary negative symptoms (curbing of interests, diminished sense of purpose, diminished social drive, restricted affect, diminished emotional range and poverty of speech) for at least 12 months, including periods of clinical stability. Positive symptoms, depression and disorganization were assessed using the Positive and Negative Syndrome Scale [81].

The daily antipsychotic dose was converted to chlorpromazine equivalents, according to Gardner et al. [84].

### 2.3. MRI Acquisition and Parameters 

We recorded all MRI with a 3 T scanner (Achieva, Philips Medical Systems, Best, The Netherlands), and we acquired DTI data using an EPI sequence (repetition time/echo time (TR/TE) 9300/102 ms, voxel 2 × 2 × 2 mm^3^, 32 directions uniformly distributed in 3-dimensional (3D) space 25, B-factors 0 and 1000 s/mm^2^, 50 axials slices covering the whole brain). In addition, we obtained a 3D T1-weighted brain volume (Turbo-Field-Echo sequence, TR/TE 7.7/3.5 ms, voxel 1 × 1 × 1 mm^3^, 181 sagittal slices covering the whole brain) to improve the spatial normalization of the data to the MNI space (see below). During the MRI acquisition, subjects were lying on their back with their heads lightly fixed by straps and foam pads to minimize head movement.

### 2.4. Region of Interest 

We choose a set of ROIs relevant to the reward system for tractographic analysis, following the approach proposed by Bracht et al. [85], integrated by a set of insular ROIs. We defined the following ROIs bilaterally as seeds: NAcc (5 mm radius sphere, MNI coordinates of the center ± 8, 11, −9) [86], Amy (as defined in the WFUPick-Atlas) [87], VTA (4 mm radius sphere, MNI coordinates of the center ±5, −20, −10) [88]. Then, we defined the following as target ROIs: mOFC, lateral orbito-frontal cortex (lOFC), DLPFC, along with ventral-anterior (vaIC), dorsal-anterior (daIC) and posterior (pIC) insular cortex.

- Left and right DLPFC were defined combining on each side the Brodmann areas 9 and 46 [89], as defined in the WFUPick-Atlas.
- Orbito-frontal cortices were preliminarily obtained by combining the Brodmann areas 10 and 11, as defined in the WFUPick-Atlas, and were then divided on each side of the brain in their medial (mOFC) and lateral (lOFC) parts using the sagittal planes placed 20 mm off-center as separators [90].
- For each side, vaIC, daIC and pIC ROIs were obtained by dividing the entire available ROIs of insular cortex in the Harvard-Oxford Cortical Structural Atlas [91], based on its connectivity [92]. DTI pre-processing and probabilistic tractography were performed using the software modules provided in the FMRIB Software Library (FSL, http://fsl.fmrib.ox.ac.uk/fsl, accessed on 15 July 2017).

### 2.5. Probabilistic Tractography

We preliminarily corrected all DTI datasets for head movements using the eddy_correct routine implemented in FSL [93], thereby correcting accordingly diffusion sensitizing gradient directions [94]. A brain mask was obtained from the B0 images using the Brain Extraction Tool routine [95], and a diffusion tensor model was fitted at each voxel using FSL’s algorithm for Bayesian Estimation of Diffusion Parameters Obtained using Sampling Techniques (BEDPOSTX). From the parameters of affine co-registration (translation along and rotation around the 3 axes), the mean movement over the brain mask was calculated for each of the 32 DTI volumes, as compared with the previous one. To avoid the effects of motion, which strongly influences apparent diffusion parameters, we excluded from the analysis datasets that exceeded at any time point 3 mm of head movement, and used mean head movement as a covariate in the second-level analysis (see below). 

Then, we normalized the deskulled B0 volumes to the MNI space using the corresponding T1-weighted volumes as a proxy, using the 152 subject T1 template provided by SPM, and the FMRIB’s Linear Image Registration Tool [96]. The resulting normalization matrices were inverted and applied to the ROIs (defined in the MNI space), to apply them to each patient’s study. We assessed visually the quality of the normalization by verifying the match between normalized B0 volumes and the EPI template provided with SPM. 

Then, we carried out probabilistic tractography using ProbTrackx [97], modeling 5000 iterations within each voxel of the seed ROI, with a curvature threshold (cosine of the minimum allowable angle between 2 steps) of 0.2, a step length of 0.5 and a maximum number of 2000 steps. For each seed–target couple, we used the percentage of the total pathways starting from the seed that reached the target as a measure of the connectivity strength between the 2 ROIs (Connectivity Index, CI). In addition, we calculated the cumulated fractional anisotropy (FA) over each pathway in order to provide a measure of its structural integrity. Given the lack of consensus on this statistical issue, we did not use a threshold for either CI or FA calculations [98].

For each seed, only connections to homolateral target ROIs were examined.

### 2.6. Statistical Analysis 

SPSS (Version 25.0, SPSS Inc, Chicago, IL, USA) was used to perform statistical analyses. A general linear model was fitted separately for each measure to assess differences between groups, including in the model as covariates age, gender and mean head movement (root mean square realignment estimates, RMS), as derived from the eddy_correct procedure. Bonferroni post-hoc comparisons between the three sample groups (HCs, DS and NDS) were performed when a significant main effect of the group emerged.

Results were considered significant for *p* < 0.05, corrected according to Bonferroni for the number of connections assessed. In particular, as only homolateral connections were examined, a total of 36 seed–target couples were tested (3 seeds × 6 targets × 2 hemispheres), so that *p* < 0.0014 was used as a statistical threshold. 

## 3. Results 

### 3.1. Subject Characteristics

We included only 46 patients and 35 HCs in the group-level analysis, as the MRI scans of six patients were discarded due to excessive motion artifacts during visual inspection. Please refer to Table S1 for the demographic and clinical characteristics of the whole sample of SCZ, as compared to HCs. 

According to the SDS criteria, the whole sample of SCZ was divided into DS (*n* = 9) and NDS (*n* = 37) patients. Table 1 summarizes the demographic and clinical characteristics of the three groups of the study sample (DS, NDS and HCs). There was no significant difference in the mean age (*p* = 0.149), gender (*p* = 0.268) or paternal (*p* = 0.057) and maternal (*p* = 0.265) education between DS, NDS and HCs. There was a small difference between the three groups in terms of RMS (*p* = 0.049). NDS, as compared to DS, had higher scores on PANSS Depression (*p* = 0.003). There was no statistically significant difference between DS and NDS on the SDS scores, although DS, as compared to NDS, had higher SDS total and subdomain scores.

### 3.2. Group Comparison on the Connectivity Index and Fractional Anisotropy between Couples of ROIs

The results of the comparison on the CI and FA between SCZ and HCs are reported in Tables S2 and S3. In particular, SCZ, as compared to HCs, had a reduced CI between rAmy and homolateral DLPFC; however, this result did not survive correction for multiple tests (*p* = 0.004) (Table S2, Figures S1 and S2).

**Figure 1:**
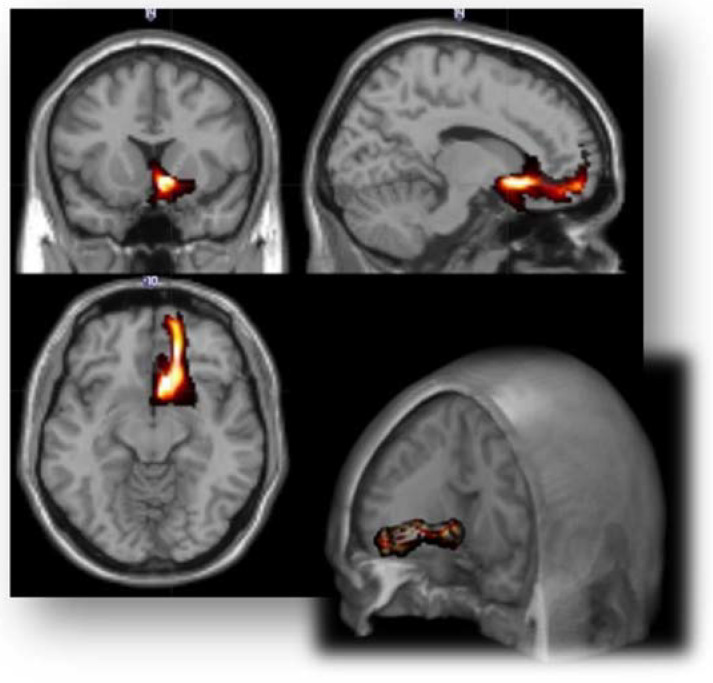
Three-dimensional representation of the average distribution of the connection patterns between the right amygdala and the ipsilateral dorsal anterior insular cortex.

**Figure 2:**
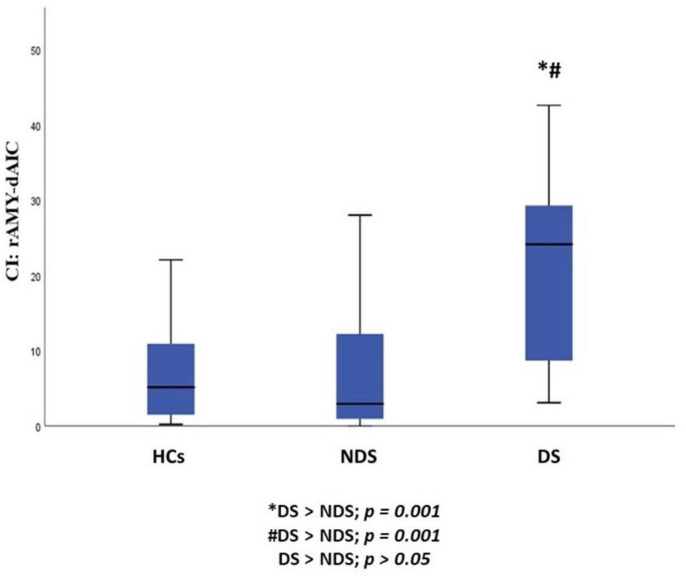
Group differences between DS, NDS and HCs in the CI of the rAmy-daIC pathway. HCs: healthy controls; DS: subjects with deficit schizophrenia; NDS: subjects with non-deficit schizophrenia CI: connectivity index; rAmy: right amygdala; daIC: dorsal-anterior insular cortex.

When we compared the three sample groups (DS, NDS and HCs), we observed a statistically significant difference in CI in the rAmy-daIC pathway (*p* = 0.001). Post hoc pairwise comparisons demonstrated that DS, as compared to NDS (*p* = 0.001) and HCs (*p* = 0.001), showed an increase in CI in the rAmy-daIC pathway, while no statistically significant difference was found between NDS and HCs (Table 2, Figure 1 and Figure 2).

**Figure 3:**
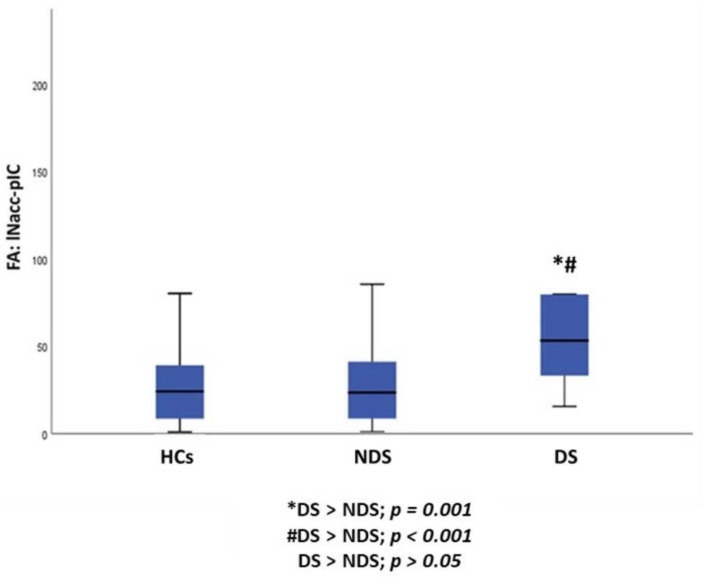
Group differences between DS, NDS and HCs in the FA of the lNAcc-pIC pathway. HCs: healthy controls; DS: subjects with deficit schizophrenia; NDS: subjects with non-deficit schizophrenia FA: fractional anisotropy; lNAcc: left nucleus accumbens; pIC: posterior insular cortex.

Furthermore, a statistically significant difference between DS, NDS and HCs was observed in FA of the lNAcc-pIC pathway (*p* = 0.001). Post hoc pairwise comparisons demonstrated an increase in FA of the lNAcc-pIC pathway in DS compared to both NDS (*p* = 0.001) and HCs (*p* < 0.001), while no differences were found between NDS and HCs (Table 3, Figure 3).

Finally, the three groups differed at a trend level in the CI and FA of different pathways (Table 2). However, these results did not survive correction for multiple tests.

## 4. Discussion

In this study, we carried out a probabilistic DTI analysis to explore abnormalities in structural connectivity within motivational circuits in subjects with schizophrenia, differentiating patients with DS and NDS.

We found that all subjects with schizophrenia had a reduced CI between rAmy and homolateral DLPFC; however, this result did not survive correction for multiple tests. The altered connectivity within this circuit suggests that subjects with schizophrenia have an impairment in the integration of motivational and cognitive information for goal-directed behavior [4,39]. It is possible that the heterogeneity within the syndrome might obscure findings concerning connectivity indices within the motivational circuit.

Considering the three sample groups (DS, NDS and HCs), we found that, DS, as compared to NDS and HCs, showed 1) a significant increase in CI in the rAmy-daIC pathway and 2) a significant increase in FA of the lNAcc-pIC pathway.

According to our findings, only subjects with DS showed abnormalities in the neural pathways involving mainly the Amy, the IC and the NAcc.

Firstly, DS, in comparison to NDS and HCs, showed an increase in CI between the rAmy and the daIC. Although at a trend level, the FA of the same pathway was also increased in DS, as compared to NDS and HCs. Therefore, DS showed abnormal connectivity strength (indicated by an increased CI) and disturbed fiber integrity (indicated by an increased FA) between the amygdala and dorsal-anterior insular cortex, probably suggesting an altered pruning process [99]. Pathways connecting the amygdala and insular cortex play a critical role in modulating and mediating connections between the two motivational systems [4] and are involved in upgrading and recalling the value information to support goal-directed behavior [100,101]. In particular, the amygdala, which seems to act in close collaboration with the OFC [102,103,104,105] and the ventral and medial areas of the prefrontal cortex and ventral striatum [106,107], plays a key role in reward processing and in stimulus–reward associations [108,109,110,111,112]. It is involved in the stimulus–response association and in orienting attention towards salient stimuli, which suggests its usefulness in evaluating the environmental context [62].

As regards the daIC, several studies have suggested that this brain region plays a key role in salience processing [113] and also modulates cognitive flexibility and autonomic activation in response to environmental changes with a general recruitment of attention, executive and working memory resources [114].

Furthermore, in our work, we observed abnormalities in fiber integrity, as suggested by the increase in FA for pathways connecting the lNAcc with pIC in DS, not present in NDS and in HCs. NAcc plays a critical role in transferring information from the IC to the “associative” medial DSr and the “sensorimotor” lateral one, connected to the cortical executive circuit, to influence motivated behavior.

In addition, previous findings indicated that the NAcc-IC pathway is strongly interconnected with the social decision-making network [115], thus playing a critical role in social behaviors—for instance, social cognition, which is often impaired in subjects with schizophrenia [49,116,117,118]. The IC is a site of multisensory integration [119,120,121] that provides an important cortical input to the NAcc, involved in reward [122,123]. Abnormalities in pathways connecting the lNAcc with pIC in DS observed in our study might be interpreted in light of the presence in DS of a greater impairment of social cognition, in comparison with NDS and HCs [21,22,23,24,25,26,27,28,29,30].

Overall, our results could be interpreted in light of previous observations in animal studies. For instance, as has been demonstrated in rodents, the connections of IC with the basolateral amygdala (BLA) and NAcc within the motivational pathways are involved in the dynamic adjustment of behavior with respect to changes in outcome valuation, depending on the current motivational state (e.g., reduced motivation to look for a drink when not thirsty), an important aspect of motivation to engage in goal-directed behavior. BLA and IC give rise to a circuit in which BLA encodes and upgrades changes in outcome value, while IC, due to its connections with the NAcc, plays a key role in retrieving the encoded changes in outcome values to direct choices between motivated actions [100,101]. Therefore, our findings seem to highlight that a dysfunction within the motivational salience circuit and impaired connections between brain regions (Amy and IC) that serve as an interface between the two motivational circuits are fundamental aspects of DS. The structural hyperconnectivity found in these subjects might be interpreted as an altered pruning process in cerebral regions devoted to updating the value that a stimulus has for a subject to support goal-directed behavior [4,39,40,99].

Our study has several strengths. Indeed, previous studies that investigated WM alterations in DS did not search for abnormalities of motivational circuits, since this was not the primary objective of these studies [4,39,40,75,76,77,78,79,80]. Furthermore, in our study, the assessment of deficit schizophrenia was made using the SDS, which is regarded as the gold-standard instrument in this field. In some of the previously mentioned studies [77,79,80], deficit schizophrenia was assessed using a proxy derived from the PANSS. The latter method for categorizing patients as DS and NDS has some problems in terms of face validity and temporal stability [14].

Structural connectivity analysis, which is used in this study, is not affected by poor general intellectual abilities or memory impairment, often present in subjects with schizophrenia, as subjects do not have to perform a task.

Our findings should be also interpreted in light of some limitations. First, the sample size is relatively small, which limits the possibility of generalizing the results. The small number of DS included in the analysis could prevent the detection of significant results. Further studies with larger samples, including a higher number of DS, are needed. In addition, the use of the SDS has prevented the evaluation of the severity of negative symptoms and testing of its association with structural connectivity parameters. Indeed, the SDS was developed to categorize subjects with schizophrenia as DS and NDS, and it is not appropriate to use the scale to evaluate symptom severity. Moreover, the use of the SDS might explain why, in our study, DS did not differ from NDS in terms of negative symptom severity, since other factors are considered to differentiate DS and NDS—for instance, the distinction between primary vs. secondary negative symptoms and transient vs. enduring negative symptoms. Future studies, using both SDS and an instrument for the evaluation of negative symptom severity, are needed to test the association between the impairment in motivational circuits in DS and negative symptom severity, as well as the possible differential associations with the two negative symptom domains.

Finally, DS and NDS differed in terms of depression scores, which we could not use as a covariate in the main analysis since we did not evaluate depression in the group of healthy controls. However, we should take into account that DS, which had lower depression scores than NDS, differed in terms of structural connectivity parameters from HCs and NDS, while no difference was found between NDS and HCs. Finally, the scores of depression were very low in both patient groups, as DS had a minimal level of depression and NDS a mild level of depression, far below the threshold of clinical significance.

In conclusion, our results lend support to the hypothesis of the presence of alterations in the motivational circuits as possible pathophysiological mechanisms of negative symptoms in subjects with schizophrenia. In addition, our data support previous evidence of distinct neurobiological alterations underlying the different clinical subtypes of schizophrenia. In particular, subjects with deficit schizophrenia, as compared to those with non-deficit schizophrenia and to healthy controls, probably present an altered pruning process (consistent with the hyperconnectivity) in cerebral regions devoted to updating the value that a stimulus has for a subject in order to support goal-directed behavior.

## Figures and Tables

**Table 1:** Demographic characteristics, RMS and illness-related variables of the study sample (HCs, NDS and DS).

	HCs (*n* = 35)	NDS (*n* = 37)	DS (*n* = 9)	F	*p*
Age (years)	32.94 ± 8.80	36.57 ± 7.50	33.00 ± 8.53	1.952	0.149
Gender (M/F)	17/18	25/12	5/4	1.340	0.268
Paternal education (years)	11.31 ± 5.85	8.41 ± 4.64	9.00 ± 4.09	2.965	0.057
Maternal education (years)	10.34 ± 5.67	8.49 ± 4.69	8.33 ± 4.47	1.352	0.265
RMS	0.34 ± 0.10	0.41 ± 0.10	0.40 ± 0.11	3.131	0.049 *
Total SDS	-	7.82 ± 5.60	11.00 ± 6.70	1.740	0.195
SDS Experiential domain	-	4.76 ± 3.45	6.29 ± 3.20	1.161	0.288
SDS Expressive Deficit domain	-	3.06 ± 2.47	4.71 ± 3.59	2.196	0.147
PANSS Positive	-	8.09 ± 4.28	6.00 ± 2.45	1.541	0.222
PANSS Disorganization	-	7.33 ± 3.68	7.43 ± 4.28	0.004	0.952
PANSS Depression	-	2.49 ± 0.85	1.43 ± 0.50	10.224	**0.003**
Chlorpromazine equivalent doses	-	402.01 ± 190.05	263.37 ± 92.34	3.003	0.092

DS: patients with deficit schizophrenia; HCs: healthy controls; NDS: patients with non-deficit schizophrenia; PANSS: Positive and Negative Syndrome Scale; RMS: root-mean-square of the movement during the examination; SDS: Schedule for Deficit Syndrome. *p* values in boldface indicate statistical significance. * Bonferroni’s post-hoc bivariate test: DS—HCs, *p* = 0.44; NDS—HCs, *p* = 0.057.

**Table 2:** Group differences between DS, NDS and HCs in CI.

Brain Pathways	NDS (*n* = 37)	DS (*n* = 9)	HCs (*n* = 35)	F	*p*
		CI			
lNAcc to daIC	10.52 ± 14.31	6.02 ± 6.64	9.36 ± 10.88	0.831	0.440
lNAcc to DLPFC	41.35 ± 54.84	15.23 ± 19.29	49.31 ± 101.35	0.654	0.523
lNAcc to lOFC	119.56 ± 209.92	105.95 ± 116.48	120.02 ± 156.09	0.114	0.892
lNAcc to mOFC	2192.74 ± 1283.04	1975.77 ± 859.34	2903.13 ± 1783.21	2.023	0.139
lNAcc to pIC	552.43 ±591.62	1132.7 ± 850.63	463.43 ± 408.79	4.823	0.011
lNAcc to vaIC	646.07 ± 509.67	1246.7 ± 1052.93	920.0 ± 881.79	2.453	0.093
lAmy to daIC	66.28 ± 66.38	29.66 ± 24.57	69.33 ± 69.13	1.532	0.223
lAmy to DLPFC	40.26 ± 35.93	23.7 ± 17.01	53.3 ± 46.16	1.795	0.173
lAmy to lOFC	160.01 ± 175.11	74.5 ±31.49	14,328 ± 160.88	1.922	0.153
lAmy to mOFC	832.51 ±547.32	598.92 ±337.07	1012.18 ± 647.54	1.662	0.197
lAmy to pIC	2231.79 ± 1861.32	3076.22 ± 1806.24	1748.80 ± 1274.76	3.323	0.041
lAmy to vaIC	2418.53 ± 1169.48	2452.89 ± 916.74	2908.10 ± 1094.67	1.424	0.247
lVTA to daIC	57.8 ± 89.75	12.48 ± 13.78	33.06 ± 41.98	1.428	0.246
lVTA to DLPFC	125.04 ± 157.90	76.05 ± 100.19	129.57 ± 85.92	1.101	0.338
lVTA to lOFC	90.15 ± 79.93	36.76 ± 32.64	126.96 ± 140.15	2.560	0.084
lVTA to mOFC	66.57 ± 67.51	80.52 ± 153.03	102.19 ± 131.15	1.027	0.363
lVTA to pIC	76.27 ± 91.95	59.45 ± 68.97	46.88 ± 58.16	1.251	0.292
lVTA to vaIC	17.21 ± 27.71	8.95 ± 3.49	16.54 ± 17.90	0.763	0.470
rNAcc to daIC	11.09 ± 44.34	12.36 ± 14.58	7.99 ± 16.06	0.088	0.916
rNAcc to DLPFC	18.54 ± 26.02	17.21 ± 23.43	31.69 ± 59.31	0.874	0.421
rNAcc to lOFC	449.74 ± 512.68	552.32 ± 553.16	583.00 ± 575.02	0.099	0.906
rNAcc to mOFC	1352 ± 943.98	1660.94 ± 1159.38	2216.77 ± 1315.54	3.717	0.029
rNAcc to pIC	129.18 ± 244.89	89.27 ± 110.05	80.98 ± 92.41	0.119	0.888
rNAcc to vaIC	732.42 ± 961.42	1369.91 ± 1410.50	718.38 ± 700.50	2.082	0.132
rAmy to daIC	8.39 ± 11.46	25.53 ± 21.64	7.82 ± 9.24	8.190	**0.001**
rAmy to DLPFC	20.1 ±24.27	18.14 ± 9.89	38.16 ± 32.92	4.356	0.016
rAmy to lOFC	95.41 ± 101.87	59.48 ± 39.84	129.18 ± 107.97	1.436	0.244
rAmy to mOFC	1246.66 ± 1069.13	937.87 ± 852.42	1075.12 ± 992.10	0.389	0.679
rAmy to pIC	77.06 ± 144.08	118.83 ± 138.32	70.62 ± 80.30	0.985	0.378
rAmy to vaIC	736.59 ± 824.11	818.15 ± 756.58	631.73 ± 508.82	0.502	0.607
rVTA to daIC	23.3 ± 40.78	17.45 ± 28.28	35.64 ± 59.95	0.683	0.508
rVTA to DLPFC	122.45 ± 94.52	77.53 ± 68.19	148.46 ± 124.59	2.100	0.130
rVTA to lOFC	149.33 ± 169.72	64.63 ± 82.54	134.87 ± 151.53	1.262	0.289
rVTA to mOFC	67.24 ± 93.39	26.85 ± 29.73	52.22 ± 91.28	0.656	0.522
rVTA to pIC	14.77 ± 17.26	30.91 ± 69.09	14.17 ± 18.00	1.283	0.283
rVTA to vaIC	10.68 ± 11.90	13.23 ± 17.42	17.54 ± 18.96	0.543	0.583

Amy: amygdala; CI: connectivity index; daIC: dorsal-anterior insular cortex; DLPFC: dorso-lateral prefrontal cortex; HCs: healthy controls; l: left; lOFC: lateral orbito-frontal cortex; mOFC: medial orbito-frontal cortex; Nacc: nucleus accumbens; pIC: posterior insular cortex; r: right; SCZ: subjects with schizophrenia; vaIC: ventral-anterior insular cortex; VTA: ventral tegmental area. *p* < 0.0014 was used as statistical threshold; *p* values in boldface indicate statistical significance corrected for multiple tests; Bonferroni’s post-hoc bivariate test: CI rAmy to daIC: DS—NDS, *p* = 0.001; DS—HCs, *p* = 0.001.

**Table 3:** Group differences between DS, NDS and HCs in FA.

Brain Pathways	NDS (*n* = 37)	DS (*n* = 9)	HCs (*n* = 35)	F	*p*
		FA			
lNAcc to daIC	1.54 ± 1.27	1.26 ± 0.81	1.60 ± 1.20	0.273	0.762
lNAcc to DLPFC	3.35 ± 3.76	1.69 ± 1.25	3.27 ± 3.55	0.967	0.385
lNAcc to lOFC	7.14 ± 10.8	6.45 ± 4.54	7.32 ± 7.66	0.081	0.923
lNAcc to mOFC	49.93 ± 29.7	61.72 ± 41.81	63.01 ± 46.42	0.857	0.429
lNAcc to pIC	31.77 ± 27.32	76.48 ± 68.31	28.20 ± 22.57	7.760	**0.001**
lNAcc to vaIC	27.45 ± 22.09	52.3 ± 39.84	29.48 ± 20.19	4.202	0.019
lAmy to daIC	6.85 ± 5.22	4.01 ± 1.93	6.78 ± 5.80	1.645	0.200
lAmy to DLPFC	1047.97 ± 167.3	1099.55 ± 207	1030.49 ± 232.41	0.347	0.708
lAmy to lOFC	0.99 ± 1.36	0.57 ± 0.32	0.86 ± 0.91	0.792	0.457
lAmy to mOFC	12.80 ± 17.30	5.14 ± 5.73	6.74 ± 9.25	0.935	0.397
lAmy to pIC	51.63 ± 36.29	74.7 ± 31.73	45.06 ± 27.15	3.351	0.040
lAmy to vaIC	43.46 ± 17.84	43.71 ± 14.56	51.67 ± 22.79	2.098	0.130
lVTA to daIC	5.00 ± 5.80	2.11 ± 0.71	4.12 ± 3.99	1.079	0.345
lVTA to DLPFC	8.29 ± 8.86	5.84 ± 5.69	8.36 ± 4.55	0.848	0.432
lVTA to lOFC	8.53 ± 6.07	5.16 ± 4.09	10.04 ± 8.15	1.982	0.145
lVTA to mOFC	6.11 ± 4.86	5.24 ± 5.8	8.04 ± 6.98	1.967	0.147
lVTA to pIC	5.99 ± 5.42	4.66 ± 3.48	3.76 ± 3.35	2.324	0.105
lVTA to vaIC	1.96 ± 1.45	1.62 ± 0.5	2.01 ± 1.12	0.389	0.679
rNAcc to daIC	1.58 ± 3.42	2.13 ± 1.72	1.14 ± 0.94	0.540	0.585
rNAcc to DLPFC	2.20 ± 2.93	1.67 ± 1.47	2.82 ± 4.03	0.454	0.637
rNAcc to lOFC	20.76 ± 21.26	24.71 ± 25.40	24.17 ± 20.67	0.019	0.981
rNAcc to mOFC	55.36 ± 42.34	65.68 ± 44.25	81.19 ± 54.45	1.465	0.238
rNAcc to pIC	9.65 ± 14.76	7.84 ± 8.17	7.32 ± 6.61	0.065	0.937
rNAcc to vaIC	33.99 ± 34.61	50.40 ± 39.62	26.54 ± 19.49	2.392	0.098
rAmy to daIC	2.00 ± 1.75	3.99 ± 2.16	1.85 ± 1.24	6.792	0.002
rAmy to DLPFC	17.86 ± 10.45	13.17 ± 10.71	15.21 ± 11.37	0.788	0.459
rAmy to lOFC	1.43 ± 1.53	0.65 ± 0.48	1.72 ± 2.99	1.692	0.191
rAmy to mOFC	8.49 ± 8.26	7.91 ± 7.39	9.39 ± 9.86	0.114	0.892
rAmy to pIC	8.07 ± 11.83	9.18 ± 6.32	7.21 ± 5.35	0.366	0.695
rAmy to vaIC	17.07 ± 11.95	20.13 ± 14.73	13.61 ± 7.93	1.740	0.183
rVTA to daIC	3.75 ± 7.44	2.73 ± 2.9	3.97 ± 4.86	0.214	0.808
rVTA to DLPFC	8.49 ± 5.63	5.57 ± 4.14	8.44 ± 5.63	1.222	0.300
rVTA to lOFC	12.23 ± 11.34	6.27 ± 7.3	9.24 ± 8.24	1.782	0.175
rVTA to mOFC	6.81 ± 5.86	3.36 ± 3.49	5.17 ± 8.93	0.750	0.476
rVTA to pIC	1.88 ± 1.54	2.99 ± 4.41	1.76 ± 1.26	1.437	0.244
rVTA to vaIC	1.66 ± 1.13	1.95 ± 1.64	2.10 ± 1.38	0.357	0.701

Amy: amygdala; daIC: dorsal-anterior insular cortex; DLPFC: dorso-lateral prefrontal cortex; FA: fractional anisotropy; HCs: healthy controls; l: left; lOFC: lateral orbito-frontal cortex; mOFC: medial orbito-frontal cortex; Nacc: nucleus accumbens; pIC: posterior insular cortex; r: right; SCZ: subjects with schizophrenia; vaIC: ventral-anterior insular cortex; VTA: ventral tegmental area. *p* < 0.0014 was used as statistical threshold; *p* values in boldface indicate statistical significance corrected for multiple tests; Bonferroni’s post-hoc bivariate test: FA lNAcc to pIC: DS—NDS, *p* = 0.001; DS—HCs, *p* < 0.001.

## Data Availability

All data supporting the findings of this study are available within the article and Supplementary Materials.

## Supplementary Materials

The following are available online at https://www.mdpi.com/article/10.3390/jcm11010061/s1, Table S1. Demographic characteristics, RMS and illness related variables; Table S2. Group differences between SCZ and HCs in CI; Table S3. Group differences between SCZ and HCs in FA. Figure S1. 3D representation of the average distribution of the connection patterns between the right amygdala and the ipsilateral dorso-lateral prefrontal cortex; Figure S2. Group differences between SCZ and HCs in the CI of the pathway connecting right amygdala and the ipsilateral dorsolateral prefrontal cortex.
