# Supplementary material for: Investigating the Relationship between White Matter Connectivity and Motivational Circuits in Subjects with Deficit Schizophrenia: A Diffusion Tensor Imaging (DTI) Study
Source: J Clin Med. 2021 Dec 23;11(1):61. doi: 10.3390/jcm11010061 (PMC8745695; doi:10.3390/jcm11010061)
Supplement: Supplementary file 1 [file jcm-11-00061-s001.zip › jcm-1505102-supplementary.pdf]

Supplementary Materials

Table S1. Demographic characteristics, RMS and illness related variables.

	SCZ (n = 46)	HCs (n = 35)	F	p
Age (years)	35.87 ± 7.74	32.94 ± 8.80	2.521	0.116
Gender (M/F)	30/16	17/18	2.269	0.136
Paternal education (years)	8.52 ± 4.50	11.31 ± 5.85	5.901	**0.017**
Maternal education (years)	8.46 ± 4.59	10.34 ± 5.67	2.732	0.102
RMS	0.40 ± 0.11	0.34 ± 0.10	6.339	**0.014**
Total SDS	8.55 ± 6.04			
SDS Experiential Domain	5.16 ± 3.53			
SDS Expressive Deficit	3.39 ± 2.79			
PANSS Positive	7.73 ± 4.02			
PANSS Disorganization	7.29 ± 3.70			
PANSS Depression	2.31 ± 0.89			
Chlorpromazine equivalent doses	379.52 ± 184.30			

HCs: healthy controls; PANSS: Positive and Negative Syndrome Scale; RMS: root-mean-square of the movement during the examination; SCZ: subjects with schizophrenia; SDS: Schedule for the Deficit Syndrome. p values in boldface indicate statistical significance.

Table S2. Group differences between SCZ and HCs in CI.

Brain Pathways	SCZ (n = 46)	HCs (n = 35)	F	p
		**CI**		
lNAcc to daIC	9.64 ± 13.23	9.36 ± 10.88	0.174	0.678
lNAcc to DLPFC	36.23 ± 50.80	49.31 ± 101.35	0.144	0.706
lNAcc to lOFC	116.90 ± 194.15	120.02 ± 156.09	0.035	0.851
lNAcc to mOFC	2150.29 ± 1206.57	2903.13 ± 1783.21	3.897	0.052
lNAcc to pIC	665.960 ± 680.30	463.43 ± 408.79	2.261	0.137
lNAcc to vaIC	763.58 ± 680.40	920.90 ± 881.79	0.485	0.488
lAmy to daIC	59.12 ± 62.03	69.33 ± 69.13	0.721	0.398
lAmy to DLPFC	37.02 ± 33.59	53.3 ± 46.16	1.536	0.219
lAmy to lOFC	214.58 ± 194.25	143.28 ± 160.88	1.268	0.264
lAmy to lOFC	786.81 ± 518.29	1012.18 ± 647.54	1.974	0.164
lAmy to pIC	2397.00 ± 1861.81	1748.80 ± 1274.76	5.031	0.028
lAmy to vaIC	2425.25 ± 1115.23	2908.10 ± 1094.67	2.880	0.094
lVTA to daIC	48.93 ± 82.51	33.06 ± 41.98	0.189	0.665
lVTA to DLPFC	115.45 ± 148.71	129.57 ± 85.92	0.887	0.349
lVTA to lOFC	79.70 ± 75.88	126.96 ± 140.15	3.299	0.073
lVTA to mOFC	69.30 ± 88.54	102.19 ± 131.15	2.018	0.159
lVTA to pIC	72.98 ± 87.49	46.88 ± 58.16	2.351	0.129
lVTA to vaIC	15.60 ± 25.0	16.54 ± 17.90	0.044	0.835
rNAcc to daIC	11.34 ± 40.13	7.99 ± 16.06	0.173	0.679
rNAcc to DLPFC	18.28 ± 25.30	31.69 ± 59.31	1.742	0.191
rNAcc to lOFC	469.80 ± 516.11	583.00 ± 575.02	0.159	0.691
rNAcc to mOFC	1412.45 ± 983.46	2216.77 ± 1315.54	7.012	0.010
rNAcc to pIC	121.37 ± 224.47	80.98 ± 92.41	0.013	0.909
rNAcc to vaIC	857.14 ± 1076.35	718.38 ± 700.50	0.288	0.593
rAmy to daIC	11.74 ± 15.35	7.82 ± 9.24	2.701	0.104
rAmy to DLPFC	19.72 ± 22.12	38.16 ± 32.92	8.625	0.004

rAmy to lOFC	88.38 ± 93.76	129.18 ± 107.97	1.352	0.249
rAmy to mOFC	1186.24 ± 1029.05	1075.12 ± 992.10	0.181	0.672
rAmy to pIC	85.23 ± 142.44	70.62 ± 80.30	1.007	0.319
rAmy to vaIC	752.55 ± 803.84	631.73 ± 508.82	0.999	0.321
rVTA to daIC	22.15 ± 38.45	35.64 ± 59.95	1.214	0.274
rVTA to DLPFC	113.66 ± 91.09	148.46 ± 124.59	2.691	0.105
rVTA to lOFC	132.76 ± 159.34	134.87 ± 151.53	0.029	0.864
rVTA to mOFC	59.33 ± 86.00	52.22 ± 91.28	0.206	0.651
rVTA to pIC	17.926 ± 33.60	14.17 ± 18.00	0.260	0.612
rVTA to vaIC	11.18 ± 12.97	17.54 ± 18.96	1.100	0.298

Amy: amygdala; CI: connectivity index; daIC: dorsal-anterior insular cortex; DLPFC: dorso-lateral prefrontal cortex; HCs: healthy controls; l: left; lOFC: lateral orbito-frontal cortex; mOFC: medial orbito-frontal cortex; NAcc: nucleus accumbens; pIC: posterior insular cortex; r: right; SCZ: subjects with schizophrenia; vaIC: ventral-anterior insular cortex; VTA: ventral tegmental area.

Table S3. Group differences between SCZ and HCs in FA.

**Brain Pathways**	**SCZ (*n* = 46)**	**HCs (*n* = 35)**	**F**	***p***
		**FA**		
lNAcc to daIC	1.49 ± 1.19	1.60 ± 1.20	0.051	0.822
lNAcc to DLPFC	3.03 ± 3.47	3.27 ± 3.55	0.408	0.525
lNAcc to lOFC	7.01 ± 9.85	7.32 ± 7.66	0.000	0.984
lNAcc to mOFC	52.24 ± 32.23	63.01 ± 46.42	1.290	0.260
lNAcc to pIC	40.52 ± 41.81	28.20 ± 22.57	2.373	0.128
lNAcc to vaIC	32.31 ± 27.78	29.48 ± 20.19	0.315	0.576
lAmy to daIC	6.29 ± 4.88	6.78 ± 5.80	1.309	0.256
lAmy to DLPFC	1058.06 ± 174.46	1030.49 ± 232.41	0.394	0.532
lAmy to lOFC	0.91 ± 1.24	0.86 ± 0.91	0.100	0.753
lAmy to mOFC	11.30 ± 15.96	6.74 ± 9.25	0.660	0.419
lAmy to pIC	56.14 ± 36.30	45.06 ± 27.15	3.298	0.073
lAmy to vaIC	43.51 ± 17.10	51.67 ± 22.79	4.251	0.043
lVTA to daIC	4.43 ± 5.32	4.12 ± 3.99	0.107	0.745
lVTA to DLPFC	7.81 ± 8.34	8.36 ± 4.55	0.636	0.428
lVTA to lOFC	7.87 ± 5.85	10.04 ± 8.15	2.266	0.136
lVTA to mOFC	5.94 ± 5.00	8.04 ± 6.98	3.787	0.055
lVTA to pIC	5.73 ± 5.10	3.76 ± 3.35	4.406	0.039
lVTA to vaIC	1.89 ± 1.32	2.01 ± 1.12	0.003	0.956
rNAcc to daIC	1.69 ± 3.15	1.14 ± 0.94	0.727	0.397
rNAcc to DLPFC	2.09 ± 2.70	2.82 ± 4.03	0.714	0.401
rNAcc to lOFC	21.54 ± 21.88	24.17 ± 20.67	0.001	0.976
rNAcc to mOFC	57.38 ± 42.42	81.19 ± 54.45	2.762	0.101
rNAcc to pIC	9.29 ± 13.66	7.32 ± 6.61	0.029	0.866
rNAcc to vaIC	37.20 ± 35.79	26.54 ± 19.49	2.084	0.153
rAmy to daIC	2.39 ± 1.98	1.85 ± 1.24	1.153	0.286
rAmy to DLPFC	16.94 ± 10.55	15.21 ± 11.37	0.431	0.513
rAmy to lOFC	1.28 ± 1.42	1.72 ± 2.99	2.611	0.110
rAmy to mOFC	8.38 ± 8.02	9.39 ± 9.86	0.220	0.640
rVTA to daIC	3.55 ± 6.78	3.97 ± 4.86	0.231	0.632
rVTA to DLPFC	7.92 ± 5.46	8.44 ± 5.63	0.476	0.492
rVTA to lOFC	11.06 ± 10.87	9.24 ± 8.24	0.475	0.493
rVTA to mOFC	6.14 ± 5.62	5.17 ± 8.93	0.123	0.727
rVTA to pIC	2.10 ± 2.36	1.76 ± 1.26	0.488	0.487

rVTA to vaIC	1.71 ± 1.23	2.10 ± 1.38	0.669	0.416

Amy: amygdala; daIC: dorsal-anterior insular cortex; DLPFC: dorso-lateral prefrontal cortex; FA: fractional anisotropy; HCs: healthy controls; l: left; lOFC: lateral orbito-frontal cortex; mOFC: medial orbito-frontal cortex; NAcc: nucleus accumbens; pIC: posterior insular cortex; r: right; SCZ: subjects with schizophrenia; vaIC: ventral-anterior insular cortex; VTA: ventral tegmental area.

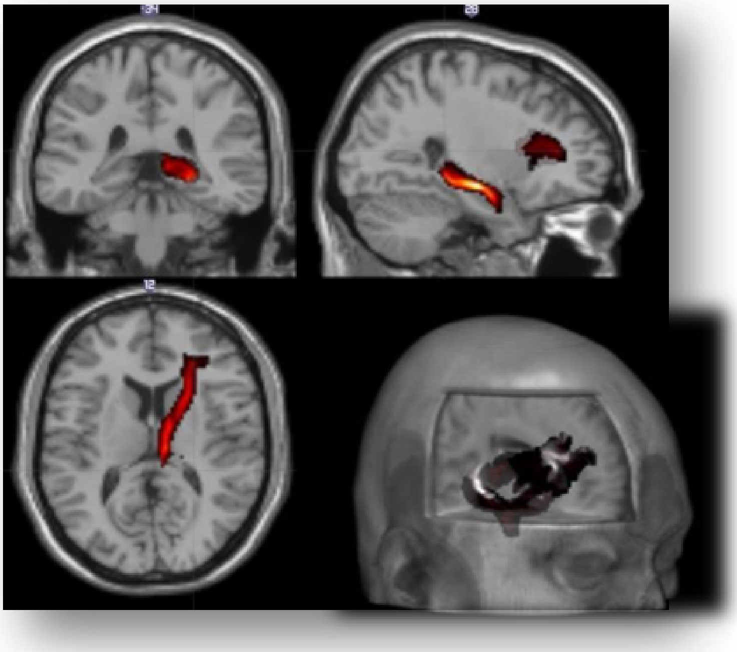

Figure S1. 3D representation of the average distribution of the connection patterns between the right amygdala and the ipsilateral dorsolateral prefrontal cortex.

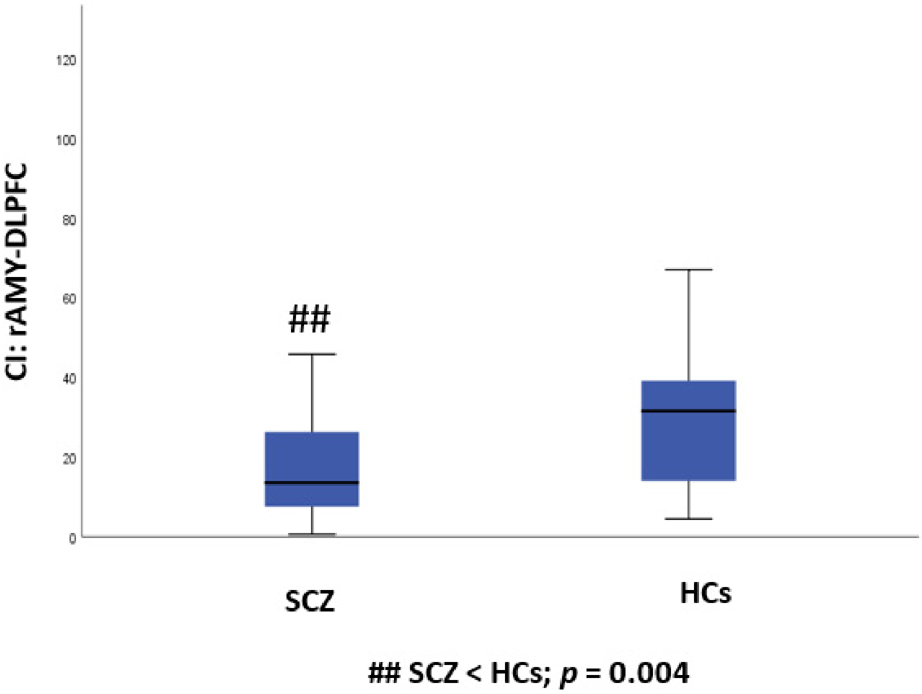

Figure S2. Group differences between SCZ and HCs in the CI of the pathway connecting right amygdala and the ipsilateral dorsolateral prefrontal cortex. **CI:** Connectivity index; **rAmy:** right amygdala; **dlPFC:** dorsolateral prefrontal cortex; **SCZ:** subjects with schizophrenia; **HCs:** healthy controls.
